# Biogas production from thermochemically pretreated sweet potato root waste

**DOI:** 10.1016/j.heliyon.2022.e10376

**Published:** 2022-08-24

**Authors:** Chebet Catherine, Maurice Twizerimana

**Affiliations:** a Department of Manufacturing, Industrial and Textile Engineering, School of Engineering, Moi University, Eldoret, Kenya; b Africa Center of Excellence II in Phytochemicals, Textiles and Renewable Energy (ACE II PTRE), Moi University, Eldoret, Kenya

**Keywords:** Pre-treatment, Biogas, Sweet potato waste, Anaerobic digester, Thermochemical pre-treatment

## Abstract

This paper presents the results of batch anaerobic digestion (AD) of thermochemically pre-treated sweet potato root waste (SPW). This agricultural waste is available in massive quantities yet it has remained an unexploited resource amid the ever-increasing need for clean energy and waste disposal challenges. Therefore, the waste can be considered for energy production through AD. However, SPW has a complex amylopectin structure that is resistant to digestive enzymes during hydrolysis which could lead to a longer retention time in the digester. In this sense, the effect of thermochemical pre-treatment on biogas production from SPW was investigated by pre-treating the substrate with sodium hydroxide (NaOH) at (0.6 g/L–3.5 g/L), temperature (50 °C–90 °C) and pretreatment time (30–120 min). The central composite design was used to design the number of experiments. SPW was milled to a small size. The physicochemical characteristics of materials were determined using standard methods. The quality of biogas produced in terms of methane content was analysed. The results from the study revealed that thermochemical pre-treatment on SPW improved biogas and methane yields. The pre-treated SPW had superior results to the untreated one. It represented a 33.88% improvement from 28.23 mL/gSPW biogas yield for the untreated SPW to 37.8 mL/gSPW for the treated SPW at optimal conditions. The optimum conditions for biogas production were found at a NaOH concentration of 2.9 g/L, a heating temperature of 82 °C, and a pre-treatment time of 102 min. Methane content in the biogas also improved from 42% to 64% (22% increase). The digester retention time was also reduced from 22 to 16 days. It can therefore be concluded that thermochemical pre-treatment of SPW improves both biogas yield and methane content as well as improves the kinetics of AD.

## 1 Introduction

Energy is one of the most essential factors for growth in all aspects of any nation (Gopinatthan et al., 2015; Jena et al., 2017). The global energy requirement has been growing at an unexpected rate (Deressa et al., 2015) due to human population growth, industrialization, and transportation.

The energy needs are met by three main energy sources which are petroleum, gas, and coal, which together supply approximately 82–88% of the global energy requirement (Browne and Murphy, 2013; Gopinatthan et al., 2015; Schweinberger et al., 2016). In Kenya for instance, the energy sector also relies on three main sources of energy: biomass, petroleum, and electricity, at 68%, 21%, and 9% respectively. Therefore biomass is the Country’s major source of energy from wood-burning and charcoal (IEA - Kenya, 2015). The use of fossil fuel as the main source of energy has raised a great extent of concerns which include; fuel reservoir depletion which threatens its future supply, the emission of greenhouse gases which are harmful to both environment and human health as well as the high cost of the fuel resource amongst others.

To alleviate the negative impacts caused by fossil fuels, active extensive research for more renewable energy sources has become a top priority in most countries (Cesaro and Belgiorno, 2015; Vindis et al., 2009). Renewable energy is an energy source that is provided naturally, it is often acquired from the sun or natural movements and mechanisms of the environment (Cucchiella and Adamo, 2013). Bioethanol, biogas, and biodiesel are produced on large scale for commercial purposes (Comparetti et al., 2017). The utilization of biomass like crop residue, textile wool, lignocellulosic waste, industrial garbage, agricultural wastes, and food wastes as a source of renewable energy has attracted a great deal of attention because it’s an economically sustainable technology that meets the energy needs and contributes to environmental protection (Ulises et al., 2019).

In Kenya, sweet potato (SP) is a staple crop that is grown in 43 out of 47 counties. A total of l,763,643 tons of SP were produced in the year 2014 from 61,067 ha (Abong et al., 2016). Nationally the annual production of the SP has been expanding over the years, this increase is attributed to farmers slowly shifting to the crop for various reasons like; pest and dieses attacks on major crops such as maize, decreasing soil fertility caused by wrong farming practices, and a growing understanding by consumers that SP is a healthy crop and not a poor man’s crop (Fatunbi, 2018). As an example, in Bomet County, the crop has gained huge acceptance and its production has grown suddenly between the years 2012–2014, where a rise in SP production from 4650 tons to 30,971 tons was recorded (MoALF, 2018). In the opinion of Nzila et al. (2015), Kenya produces agricultural waste in massive quantities that are unexploited and when the waste is cast out using conventional methods like burning, it leads to environmental pollution. Therefore, anaerobic digestion (AD) of agricultural residues like sweet potato tuber wastes (SPW) to supply methane is the best organic waste disposal method at the same time generating energy for domestic use (Gopinatthan et al., 2015; Nzila et al., 2015).

Anaerobic digestion is a complex organic process operated by various groups of microorganisms that convert organic matter to biogas through four major steps, including hydrolysis, acidogenesis, acetogenesis, and methanogenesis (Khalid et al., 2011). The second and third steps are called acid formation steps and the fourth one is termed the methane formation stage. The time taken for the complete conversion of organic matter to biogas depends on the chemical bonding of the carbohydrates within the biomass (Bochmann and Montgomery, 2013). A bottleneck step among these steps is hydrolysis where a complex molecule of organic waste is broken into monomers, this step takes the longest time (Kasper and Schiffels, 2016). However, Zheng et al. (2014) reported that methanogenesis is regarded as a rate-limiting step within the AD process due to the slowest growth of methanogens as well as their high sensitivity to temperature, pH variations, and inhibitors. Generally, biogas is often produced from any organic matter (Bochmann and Montgomery, 2013; Deressa et al., 2015; Horváth and Taherzadeh, 2016). However, some substrates might not be suitable for biogas production for reasons such as; the substrate might be having a complex molecular structure, be highly crystalline, or is lignin-rich hence it’s poorly accessible by microorganisms and their enzymes; substrate may contain chemicals that inhibit growth and biological activity of microorganisms; the feedstock might be light hence float within the digester causing physical problems like blockages in biogas plants (Drosg et al., 2013; Horváth and Taherzadeh, 2016; Montgomery et al., 2015; Wang et al., 2018). Sometimes all the problems exist together.

SPW is available in massive quantities as the waste is generated throughout the supply chain of SP. Globally, it is estimated that SP waste generated annually ranges from 5% to 7% of the total production which amounts to approximately 5 million metric tons of waste that currently has little commercial utilization (Makini et al., 2018). The waste is rich in high-energy carbohydrates hence suitable as a feedstock for biofuel production (Felipe, 2018; Ojewumi et al., 2018; Schweinberger et al., 2016). However, SPW is rather recalcitrant and consequently not directly suitable for AD since the SP starch granules have a double crystalline structure which is complex and resistant to digestive enzymes during hydrolysis compared to cereals (Mussoline and Wilkie, 2015). Also according to Duvernay (2008) and Ojewumi et al. (2018) long and complex amylopectin chains in root and tuber starch make it difficult to hydrolyse into organic matter, hence might lead to prolonged hydrolysis leading to longer retention time in the digester. Pre-treatment before AD can help solve the digestion barriers (Bochmann and Montgomery, 2013; Taherzadeh and Karimi, 2008a). There are several pre-treatment methods for biogas feedstock which are broadly classified into four methods; mechanical, chemical, biological, and hybrid methods (Salelign and Duraisamy, 2021; Taherzadeh and Karimi, 2008b). Choosing an appropriate method of pre-treatment for a biogas feedstock is of great importance since each pre-treatment method produces different effects on the substrate (Gillian, 2011). The major goals of the pre-treatment are to disorganize the crystalline structure of micro-and macro-fibrils hence improving accessibility of soluble organic materials and to alter the pores for microbial breakdown. This results in an increase in the rate of biomass degradation by speeding up the hydrolysis phase which takes the longest time among AD stages at the same time improves biogas yield (Brodeur et al., 2011; Mu and Zhang, 2019; Sindhu et al., 2015). An increase in biogas production and a reduction in the incubation time of substrate within the digester are the two most vital factors in biogas production which should be put into consideration (Haghighat et al., 2019). Frigon and Guiot (2010) reported that pre-treatments of sugar and starch crops are rarely mentioned because these substrates are easily biodegradable. However, pre-treatment is necessary since it produces positive results in methane yield and incubation time.

According to a comparative analysis studied by Felipe (2018), cumulative biogas produced varies with SP genotypes varies. Akoetey et al. (2017) also compared biogas production from tropical forestry (albizzia) wastes with food wastes including SP, taro, and papaya. They observed that the highest biogas yield was from waste that had SP with values ranging from 371 to 411 L kg/VS of CH_4_ yields. Montoro et al. (2019) co-digested SP and dairy cow manure and observed that increasing the proportion of SP from 0 to 50% in co-digestion with dairy cow manure increased biogas and methane yield. Martins et al. (2019) also co-digested poultry slaughter wastewater and SP, they found that the highest methane yield was obtained when poultry slaughtering waste was at 80% while the SP was at 20%. Above this ratio, biogas production ceased after seven days. According to Akoetey et al. (2017), biogas produced from the AD of SPW can be used to offset energy requirements in a processing plant. Even though a great extent of research has been done on SP as a potential source of renewable energy, to the best of our knowledge, no previous study has been done to investigate the effects of pre-treatment on sweet potato waste for biogas production. Therefore, this study aimed to investigate the effects of thermochemical pre-treatment on biogas production from SPW.

## 2 Materials and methods

### 2.1 Substrate preparation and quantification of waste generated from manual peeling

**Figure 1:**
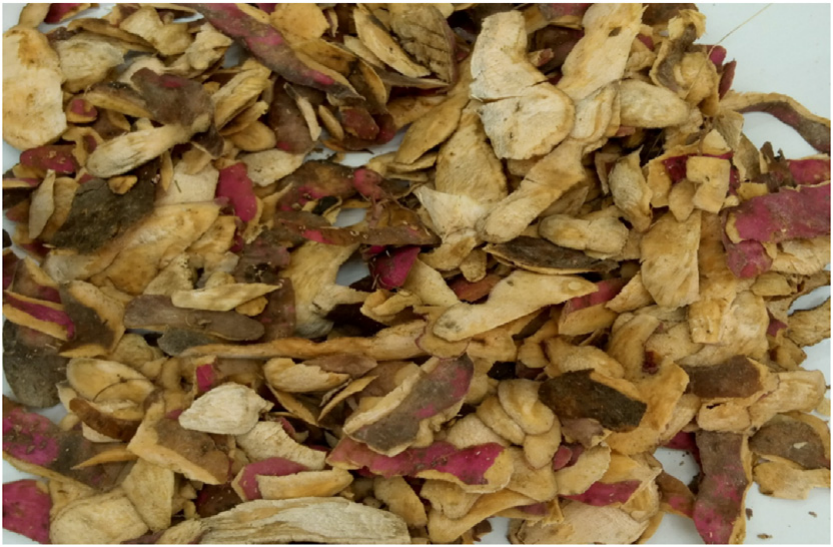
Cleaned SP from manual peeling.

The experimental studies in this research were conducted between November 2019–December 2020 in Moi University Laboratories in Eldoret and partly in Kenya Agricultural and Livestock Organization (KALRO) in Kabete, Kenya. The SP was purchased from a local farm in Bomet County. Sweet potatoes were washed thoroughly with tap water to remove all the adhering soil, dirt and impurities then left to drain for 1 h. The cleaned SP were weighed and then subjected to manual peeling using a sharp knife to generate peels as shown in Figure 1.

**Figure 2:**
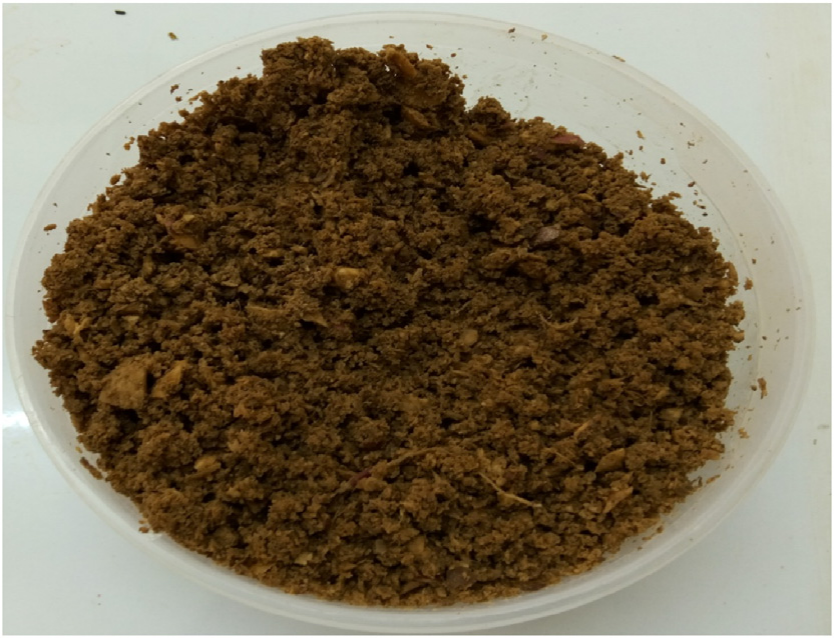
Milled SPW.

The generated waste was subjected to size reduction shown in Figure 2 using a laboratory blender (NUTRIBULLET 600 series). SPW was subjected to size reduction by the use of a laboratory blender for 1 min. This was to increase the surface area for faster degradation.

### 2.2 Physicochemical characterization of sweet potato waste

The biogas production from any substrate is extremely dependent on the carbon to nitrogen (C/N) ratio of the material, pH, temperature, total solids (TS), and volatile solids (VS) (Dioha et al., 2013). The pH measurements were performed using a bench pH meter. TS, VS, and fixed solids (FS) were determined by gravimetric methods based on the drying and the ignition of the sample (Hasanzadeh et al., 2018). For macronutrients determination, SPW was tried at 65 °C in a forced air oven until a constant weight was achieved, ground in a cutting mill, and all the organic matter in the SPW was digested completely in a Digesdahl Hach digester using a mixture of sulfuric acid (H_2_SO_4_) and hydrogen peroxide (H_2_O_2_) at 50%. The amount of potassium and phosphorus in the digestion extract was determined using the methods described by Smith (2016), Thiex (2019) and Wieczorek et al. (2022) whereby phosphorus levels were determined by the colorimetric method using a spectrophotometer (model DR-2000). The potassium content was determined using an atomic absorption spectrophotometer (model GBC 932 AA).

The carbon concentration was determined calorimetrically by using a spectrophotometer at 600 nm. Nitrogen was determined using a micro-Kjeldahl distillation unit using the standard method (APHA, 2012).

### 2.3 Effects of thermochemical pre-treatment on SPW

To examine the combined effects of the pre-treatment factors: pre-treatment time in minutes (min), the temperature in degree Celsius (°C) and sodium hydroxide (NaOH) concentration (Con.) in gram per litter (g/L), Central Composite Design in Minitab 17 software was employed to design experiments of three factors and five levels as shown in Table 1.

**Table 1:** Independent variables with their level codes.

FACTORS	CODED LEVELS				
	-2	-1	0	+1	+2
Temperature (°C)	50	58	70	82	90
Time (minutes)	30	49	75	102	120
NaOH. Con. (g/L)	0.6	1.2	2.1	2.9	3.5

For the 3 variables; temperature, time, and NaOH concentration a total number of 20 runs were obtained by the expression 2^n^ (2^3^ = 8 factorial points), 2n (2∗3) = 6 axial points, 6 centre points of replications as given in Table 2.

**Table 2:** Design Matrix in actual values.

	Factor 1	Factor 2	Factor 3
Std	A: NaOH. Con. (g/L)	B: Temperature (°C)	C: Time (min)
1	2.1	50	75
2	1.2	58	49
3	2.9	58	49
4	1.2	58	102
5	2.9	58	102
6	2.1	70	30
7	0.6	70	75
8	3.5	70	75
9	2.1	70	75
10	2.1	70	120
11	1.2	82	49
12	2.9	82	49
13	1.2	82	102
14	2.9	82	102
15	2.1	90	75
16	2.1	70	75
17	2.1	70	75
18	2.1	70	75
19	2.1	70	75
20	2.1	70	75

Thirty grams (30 g) of milled SPW was added to a 500 mL beaker and NaOH solution was added to the beaker. Based on other previous studies of NaOH pre-treatment conducted by Chen et al. (2008) and Li et al. (2012), generally, concentrations of 3.5–5 g/L Na^+^ can moderately inhibit the activity of mesophilic methanogens whilst 8 g/L Na^+^ can cause strong inhibition.

**Figure 3:**
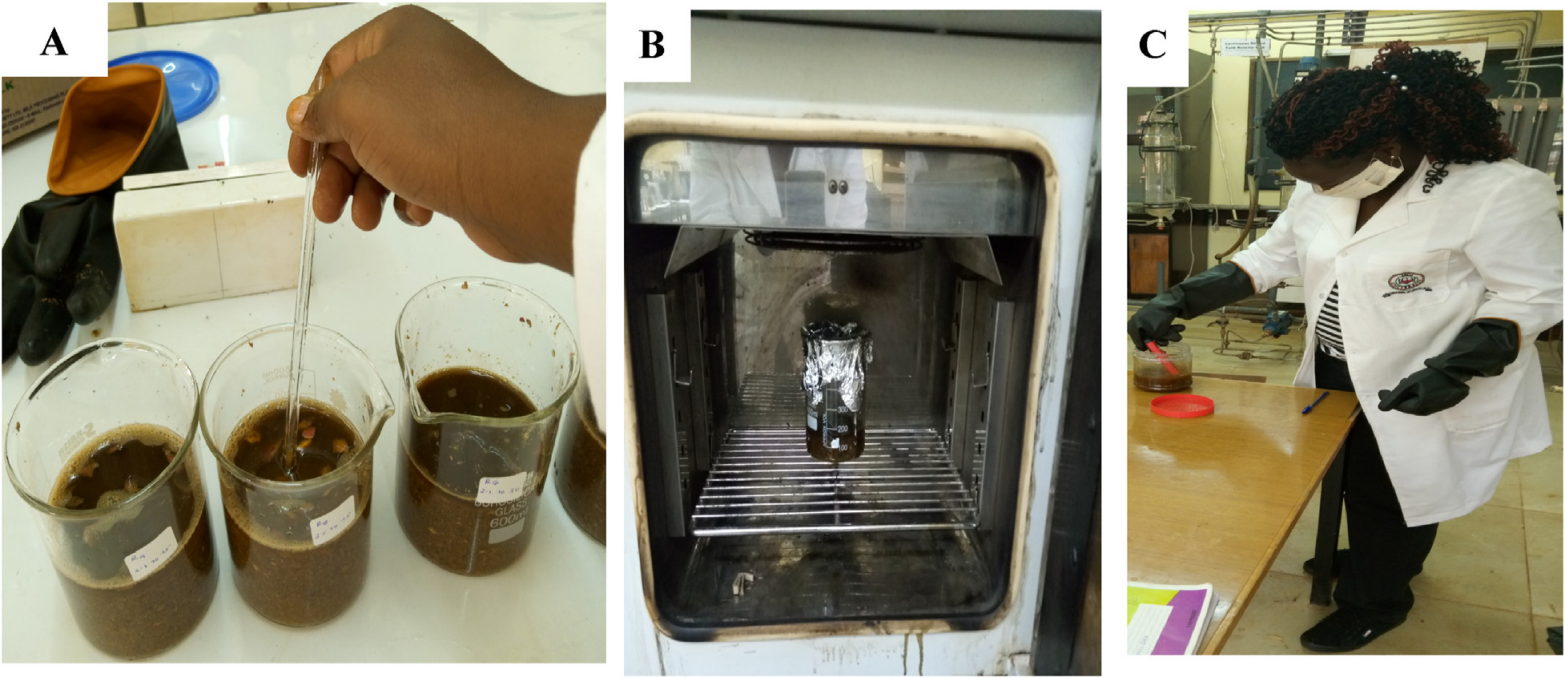
(A) Sweet potato waste suspended in NaOH solution, (B) Thermochemical pre-treatment of sweet potato waste, and (C) Neutralization of pre-treated SPW with HCl.

Therefore, the maximum NaOH concentration utilized in the present research was 3.5 g/L. The mixture was thoroughly agitated manually for 10 min as shown in Figure 3 (A) then placed in an oven at a temperature starting from 50 °C to 90 °C (Jung et al., 2015) as shown in Figure 3 (B). Thermal treatment was carried out for 30–120 min with manual shaking for 1 min every half an hour. All the pre-treated samples were adjusted to neutral pH (7.0 ± 0.2) with HCl solution as shown in Figure 3 (C) before AD and no further pH adjustment was made afterward.

### 2.4 Biochemical methane potential test (BMP)

**Figure 4:**
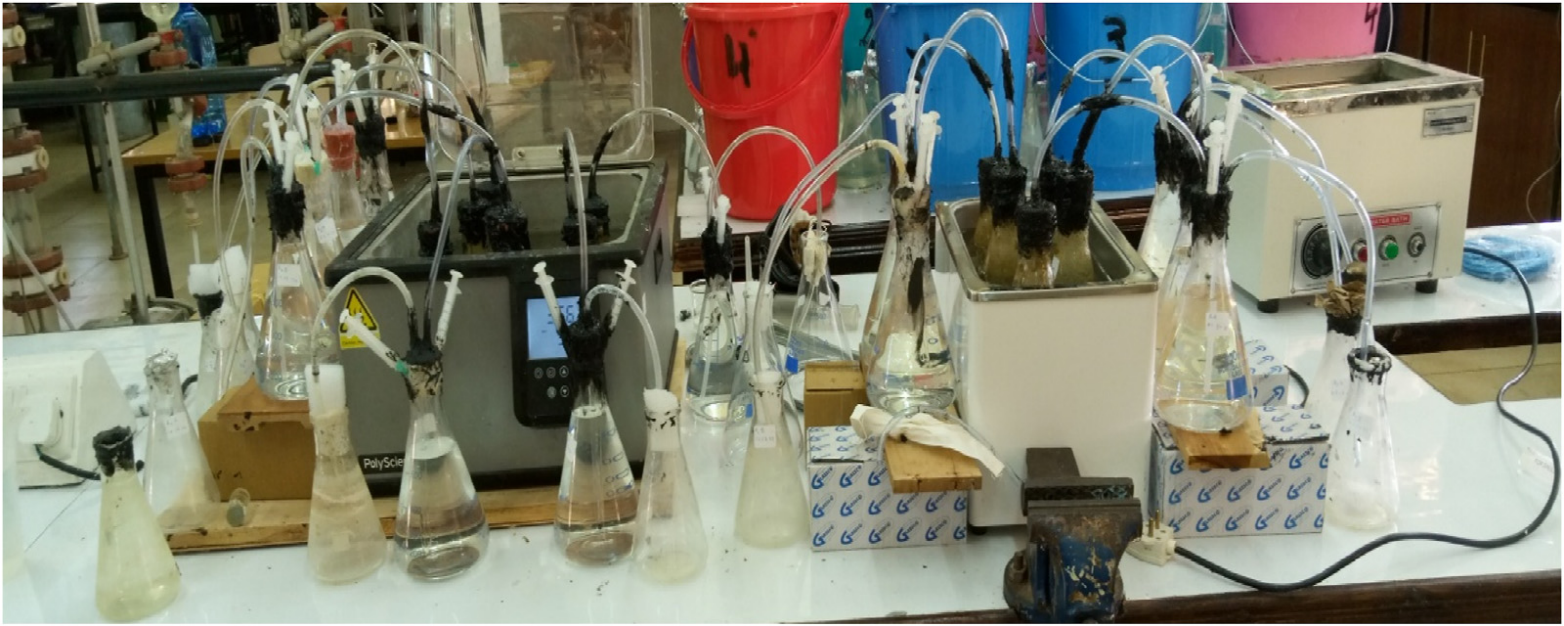
Biogas production setup.

Biochemical methane potential (BMP) tests were performed to investigate the effect of NaOH and thermal pre-treatment on biogas production from pre-treated SPW, the setup was set based on the methodology described by Braun (2007). The batch-type digester was used because it presents the simplest form of digestion and is carried out anaerobically. 250 mL conical flasks were used as digesters, the pre-treated SPW solution was fed into the digester and mixed with active inoculum at a feedstock-inoculum (F/I) ratio of 1.2:1 based on volatile solids (VS) for inoculum and SPW were 74.9% and 96.6% respectively (Ge et al., 2014; Hossain et al., 2022; Pathak and Srivastava, 2007). Water was added to form a working volume of 150 mL, each digester was then covered with a coax then tightly sealed with silicone sealant to make it airtight and the outlet tube was connected to a gas collector which was partially filled with water. The digesters were then placed into a water bath set at 37 ± 1 °C. During the period, the digesters were shaken for 1 min every day to prevent scum formation which could inhibit biogas production. The quantity of biogas produced was measured daily through the downward displacement of the water column. The biogas production experimental setup was as shown in Figure 4.

### 2.5 Statistical analyses of results

All the experiments were duplicated in all the above analyses and the average results with ± standard deviations were presented. The Minitab version 17 software was used for the analysis of variance (ANOVA) of the data obtained from the BMP test. A confidence level of 95% was used to judge their significance. Moreover, some adequacy measures, such as R^2^, Adj-R^2,^ and Pred. R^2^ was determined to check the adequacy of the developed models. A quadratic model for biogas yield was developed.

## 3 Results and discussion

### 3.1 Quantification of waste generated during manual peeling

From manual peeling employing a sharp knife, the quantity of SPW generated based on weight was calculated and presented in Table 3.

**Table 3:** Quantity of SPW generated from manual peeling.

Sweet potato	Weight (g)	Percentage (%)
Sweet potato (before peeling)	1000	–
Sweet potato (after peeling)	809	80.9
Loss due to peeling	191	19.1

From Table 4, 19.1% of SPW was generated during manual peeling employing a knife, the result obtained is within the range of 15%–40% which was reported by Schieber et al. (2002) and Zentek et al. (2014). The fact that 1Kg of sweet potatoes produced 19.1% SPW means that in large-scale SP processing plants, a substantial amount of waste in form of peels is generated which might be used as a source of biofuel.

**Table 4:** Proximate analysis of sweet potato waste.

PARAMETER	UNIT	SPW	Inoculum
pH	pH unit	4.9 ± 0.1	7.6 ± 0.2
Moisture content (MC)	% (natural matter)	70.7 ± 2.1	89.67 ± 0.3
Total solids	% (natural matter)	28.9 ± 1.9	10.33 ± 2
Volatile solids	% of the TS	96.6 ± 1	74.9 ± 0.8
Fixed solids	% of the TS	3.4 ± 0.2	NA
Total Organic Carbon	% of the TS	38 ± 0.0	NA
Total Kjeldahl Nitrogen	% of the TS	0.93 ± 0.3	NA
C/N ratio	NA	40.86	NA
Total Potassium	g kg/TS	1.09 ± 0.0	NA
Total Phosphorus	g kg/TS	0.10 ± 0.0	NA

Values are averaged of duplicate analyses, NA: not applicable.

### 3.2 Physicochemical characterization of sweet potato waste and inoculum

Detailed characterization of biogas feedstock is of great importance to determine the suitability of a given feedstock for biogas production. Basic information such as water content, VS, and FS contents can be used to roughly determine the suitability of a given substrate for AD as well as the efficiency of the AD process (Drosg et al., 2013; Krus and Lucas, 2014; Orhorhoro et al., 2017). The physicochemical and elemental analyses of the SPW are tabulated in Table 4.

The pH in AD plays an important role since the micro-organisms involved in the process are sensitive to pH (Nzila et al., 2010). The ideal pH for AD ranges from 6.8 to 7.5 (Drosg et al., 2013). The SPW had a pH value of 4.9 ± 0.1 which was lower than the generally accepted optimum pH. Therefore, AD would be less efficient because the performance and growth of anaerobic bacteria are affected by low pH. The low pH also leads to the formation of undissociated volatile fatty acid which causes inhibition in the methanogenesis step. However, high pH values have been reported by Martins et al. (2019) and Felipe (2018) where they found that the pH of sweet potatoes were 6.20 ± 0.18 and 5.99–6.12, respectively, which was also higher than the value obtained from this study. TS and MC in a biogas feedstock are crucial to assure the balance of all AD stages in the digester (Krus and Lucas, 2014). It has been reported that the highest CH_4_ production rates occur at 60–80% of MC (Khalid et al., 2011). The MC (70.7 ± 2%) of SPW obtained from this work was therefore within the range which was reported to be ideal. Moreover, Dako et al. (2016) and Hoover (2001) reported a similar amount of MC for six sweet potato cultivars which ranged from 68.58% to 76.97% and 70% to 80% for root and tuber crops, respectively. Moisture is necessary for the growth mobility of microbes (Drosg et al., 2013); hence from SPW, a substantial amount of biogas can be produced due to sufficient moisture availability.

Volatile solid of feedstock is one of the major indicators for biogas production potential while TS is known to affect performance and the behaviour of microbial community (Yi et al., 2014). The VS 96.6 ± 1% obtained in the study indicates that SPW is rich in biodegradable organic matter thus the SPW would be expected to produce a lot of biogases if all the other factors are kept constant. The results obtained TS of 28.9% and VS of 96.6% were within the range of values VS of 96.99% and TS of 24.76% reported by Martins et al. (2019). The results were also comparable to TS and VS reported in the literature whereby Mussoline and Wilkie (2015) found that the industrial sweet potato culls had a TS of 35.5% and VS of 97.6% respectively. Waramboi et al. (2011) also reported that the 25 sweet potato cultivars in Australia had a TS ranging from 14.7% to 28%. The ash content obtained (3.4 ± 0.2%) was an indication that SPW contains inorganic matter (Ojewumi et al., 2018). The amount, however, was small and I, therefore implies that SPW is ideal for AD (Drosg et al., 2013). A similar amount of ash content was reported by Felipe (2018) from the comparative analysis of three sweet potato varieties, which ranged from 3.04 to 4.94%. Dako et al. (2016) also reported an equal range (2.78%–3.77%). However, Ivone (2015) reported a lower ash content (0.85 ± 0.08%). A sufficient amount of nitrogen in a biogas feedstock is essential for the growth of AD microbes (Drosg et al., 2013).

The SPW had total nitrogen of 0.93 ± 0.3 and total carbon of 38 ± 0.00% both based on TS which was equivalent value reported by Ivone (2015); 0.58 ± 0.08% of total nitrogen and 41.08 ± 0.32% of total carbohydrates from orange flesh sweet potato. The C/N ratio obtained from this work was 40.86 which was consistent with the C/N of 46.4:1 for sweet potatoes reported by Ge et al. (2014) and 45:1 obtained from culls of industrial sweet potatoes reported by Mussoline and Wilkie (2015). On the other hand, Martins et al. (2019) reported a much higher C/N ratio (107.80 ± 0.75). These variations could be contributed to factors such as the type of cultivar, harvesting period, soil condition, and the storage period. The recommended optimum C/N ratio of the AD substrate should be within the range of 16:1–30:1 (Gillian, 2011). This means that SPW has a higher C/N ratio. However, it has low nitrogen which is quickly consumed by AD bacteria to meet their protein requirements. Consequently, the carbon content in the SPW which would have been used to produce biogas is left out unutilised thus resulting to lower biogas production; therefore mono-digestion of SPW is inefficient for AD. Phosphorus and potassium content of SPW obtained in the research was 0.1 g kg/TS and 1.09 g kg/TS respectively. The amounts are sufficient for microbial growth. The availability of the macro-elements (NPK) in SPW means that SPW in its natural form can be used as a biofertilizer on farms.

### 3.3 Biochemical methane potential test (BMP)

**Figure 5:**
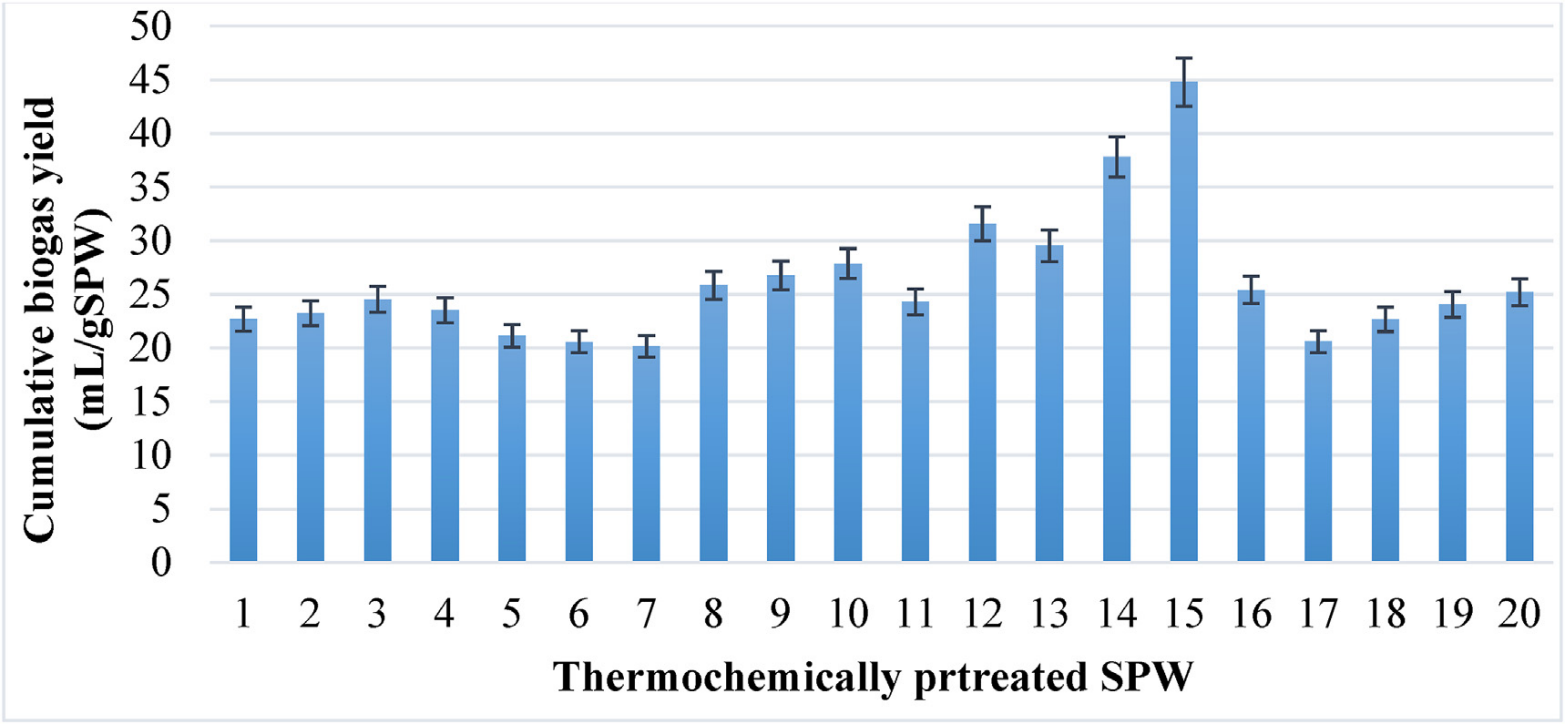
Cumulative biogas yields for the pre-treated SPW.

Thermochemical pre-treatment was tested for different NaOH concentrations, temperatures, and pre-treatment times to evaluate how pre-treatment affects biogas production from SPW. The cumulative biogas yield was as shown in Figure 5.

The quadratic model obtained from the software (Minitab 17) on biogas yield from the thermochemical pre-treated samples is as presented in Regression Equation in Uncorded.

 ​Biogas=172.4−12.79 ​Concentration−3.935 ​Temperature−0.441 ​Time ​−0.493 ​Concentration∗Concentration ​+0.02387 ​Temperature∗Temperature ​+0.000097 ​Time∗Time ​+0.2407 ​Concetration∗Temperature ​+0.0017 ​Concentration∗Time ​+0.00690 ​Temperature∗Time

The statistical model was checked by F-test and analysis of variance for the response surface quadratic model was as tabulated in Table 5.

**Table 5:** Analysis of variance.

Sources	DF	Adj. SS	Adj. MS	F-Value	p-Value
Model	6	692.991	76.999	14.17	0.000
Linear	3	428.228	142.743	26.26	0.000
Concentration	1	348.087	45.861	8.44	0.016
Temperature	1	348.087	348.087	64.05	0.000
Time	1	34.280	34.280	6.31	0.031
Square	3	177.991	59.330	10.92	0.002
Concentration∗Concentration	1	1.831	1.831	0.34	0.574
Temperature∗Temperature	1	170.254	170.254	31.33	0.000
Time∗Time	1	0.066	0.066	0.01	0.914
2-Way Interaction	3	86.772	28.924	5.32	0.019
Concentration∗Temperature	1	48.216	48.216	8.87	0.014
Concentration∗Time	1	0.011	0.011	0.00	0.965
Temperature∗Time	1	38.544	38.544	7.09	0.024
Error	10	54.350	5.435		
Lack-Of-Fit	5	30.003	6.001	1.23	0.412
Pure Error	5	24.347	4.869		
Total	19	747.341			

The overall model p-value (0.000) is less than the level of significance (0.05). Therefore, the full quadratic model of the NaOH concentration, temperature, and the time factors significantly affect the response biogas yield. The p-value for the linear terms for all the factors, the concentration, temperature, and time, are also lower than the level of significance. Therefore, the linear terms significantly affect the biogas yield. The p-value for quadratic terms for both NaOH concentration (0.574) and time (0.914) are more than the level of significance therefore the two factors are insignificant concerning biogas yield while the p-value for quadratic terms for temperature (0.000) is less than the level of significance hence temperature significantly affect the biogas yield. The interaction between the concentration and temperature (0.014) and the interaction between temperature and time (0.024) significantly affect the biogas yield.

On the other hand, the interaction between concentration and time (0.965) insignificantly affects the biogas yield. The model suffers no lack of fit because the p-value (0.412) is larger than the level of significance (0.05). Therefore, the quadratic model with the predictor variable concentration, temperature, and time significantly predicts the biogas yield. As shown in Table 7, Variance Inflation Factor (VIF) for all factors is observed to be around 1, meaning that there is no multicollinearity between a factor and the other factors. To further check how well the model fitted the data, goodness-of-fit statistics were examined in the model summary (Table 6). The coefficients S, R^2^, adjusted R^2,^ and predicted R^2^ were examined to check the model’s effectiveness. The coefficient R^2^ is the percentage of variation in the response that is explained by the model, it normally ranges between 0% and 100%. The higher the R^2^ value, the better the model fits the data, in this case, R^2^ value is 92.73% this means that the model could explain the variability of the dependent variable. The coefficient predicted R^2^ determines how well a model predicts the response for new observations. Models that have larger predicted R^2^ values have better predictive ability, in this case the value of predicted R^2^ is 63.96% this means that the model ha as 63.96% ability to predict a correct new observation. The value of adjusted R^2^ (86.18%) means that 86.18% of the variance can be predicted from the independent variable and only 13.82% of the total variation cannot be explained by the model.

**Table 6:** : Model summary.

S	R-sq	R-sq (adj)	R-sq (pred)
2.33131	92.73%	86.18%	63.96%

**Table 7:** Coded coefficients.

Term	Effect	Coef	SE Coef	T-Value	p-Value	VIF
Constant		24.117	0.951	25.36	0.000	
Concentration	3.665	1.833	0.631	2.90	0.016	1.00
Temperature	10.097	5.049	0.631	8.00	0.000	1.00
Time	3.169	1.584	0.631	2.51	0.031	1.00
Concentration∗Concentration	−0.713	−0.356	0.614	−0.58	0.574	1.02
Temperature∗Temperature	6.874	3.437	0.614	5.60	0.000	1.02
Time∗Time	0.136	0.068	0.614	0.11	0.914	1.02
Concentration∗Temperature	4.910	2.455	0.824	2.98	0.014	1.00
Concentration∗Time	0.075	0.037	0.824	0.05	0.965	1.00
Temperature∗Time	4.390	2.195	0.824	2.66	0.024	1.00

### 3.4 Response optimization of biogas

The data obtained from cumulative biogas yield presented in Table 8 was subjected to a response optimizer in Minitab 17 to determine the potential combination of the input variable settings of the three pre-treatment factors; the temperature (°C), time (min), and NaOH concentration (g/L) for optimum biogas production.

**Table 8:** Cumulative biogas yield for thermochemically pre-treated SPW and control (untreated SPW).

SN	NaOH. Con	Temperature	Time	Cumulative biogas yield (mL)
1	2.1	50	75	681
2	1.2	58	49	727
3	2.9	58	49	736
4	1.2	58	102	706
5	2.9	58	102	634
6	2.1	70	30	617
7	0.6	70	75	605
8	3.5	70	75	776
9	2.1	70	75	803
10	2.1	70	120	836
11	1.2	82	49	729
12	2.9	82	49	947
13	1.2	82	102	886
14	2.9	82	102	1134
15	2.1	90	75	1344
16	2.1	70	75	763
17	2.1	70	75	618
18	2.1	70	75	680
19	2.1	70	75	722
20	2.1	70	75	756
Control	none	none	none	847

**Figure 6:**
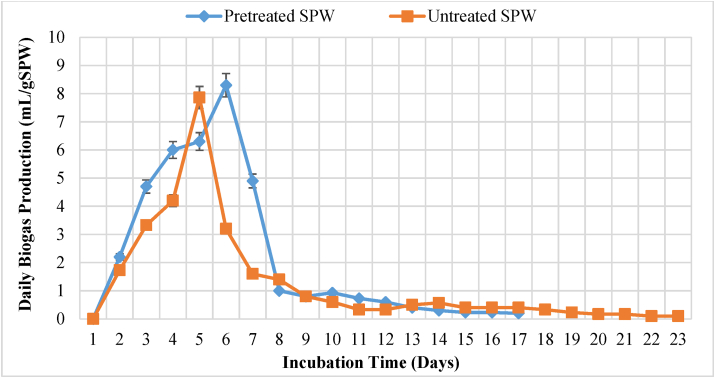
Daily biogas production.

The SPW sample pre-treated under optimal conditions was then compared with untreated SPW in terms of biogas production and methane. It was observed that in both cases, biogas production was high in the first five days and decreased after the period, as shown in Figure 6; this is due to fast digestion which may result in washout of microorganisms leading to the accumulation of intermediary products that inhibit methanogenesis step. The findings were consistent with the observation which was made by Martins et al. (2019) who reported that co-digestion of paultry sludge cake and sweet potato at S40P60, S20P80, and S0P100 produced a lot of biogas in the first seven days of operation and the production eased after that period. Tumutegyereize et al. (2016) also reported 90% methane yield in less than five days for cassava peels, sweet potato peels, and matoke peels.

Cumulatively untreated SPW produced 28.23 mL/gSPW of biogas in 22 days while the SP waste treated at 2.9 g/L NaOH, 82 °C, and 102 min produced 37.8mL/gSPW after 16 days of incubation, this represented a 33.88% improvement in biogas yield in respect to the untreated SPW. It may be seen from Table 7 that CH_4_ content in the biogas from treated samples is high compared to the untreated one. Methane content in the biogas improved from 42% to 64% (22% increase). The improvement in biogas production yield and methane content was possibly due to thermochemical pre-treatment which caused delignification of SPW and swelling of SP granules which destabilized the amylopectin crystallites facilitating enzymatic conversion of starch into sugars. Alkaline pre-treatment of the SPW could have also reduced the degree of inhibition during anaerobic digestion resulting in more biogas and methane (Chandra et al., 2012). A similar observation on temperature was made by Moorthy et al. (2012) who reported that the gelatinization temperature for two orange flesh SP varieties occurred at a temperature range of 79.27 °C–80.15 °C. Roberts and Cameron (2002) and Qin et al. (2019) also reported that the addition of NaOH to starch granules causes physicochemical changes in the structure of starch because NaOH causes sudden swelling of the granules, and application of heat on the NaOH treated starch caused further swelling leading to rupture of granules making them accessible to AD bacteria. The improvement in biogas yield could also be attributed to the alkaline nature of NaOH which caused the high solubility of SP protein-making more Nitrogen (nutrient) to be bioavailable for microbial growth (China Science Publishing and Media Ltd, 2017). The composition of biogas as obtained from a portable gas analyser was as shown in Table 9.

**Table 9:** Biogas composition.

Composition	Treated sample	Untreated sample
Methane (%)	64 ± 3.5	42 ± 2.8
Carbon Dioxide (%)	32 ± 5.6	45 ± 6.4
Hydrogen Sulphide (ppm)	142–186	156–193
Oxygen (%)	2- 4 ± 0.2	2-3 ± 0.1

Values are averaged of duplicate analyses.

The amount of methane from the untreated SP waste 42 ± 2.8% was comparable to the findings of 38.9% methane reported by Martins et al. (2019). However, a higher amount of methane ranging from 70-80% has been reported by Mussoline and Wilkie (2015).

## 4 Conclusion

The purpose of this study was to investigate the effects of thermochemical pre-treatment on biogas production from sweet potato root waste, from the findings it was concluded that; SPW which is available in large quantities in farms, markets, and sweet potato processing is rich in carbohydrates and can be utilized as a renewable energy source through the production of biogas which at the same time contributes to environmental protection. Even though sweet potato tuber waste is biodegradable, the research has indicated that thermochemical pre-treatment with NaOH at a concentration of 2.9 g/L, the temperature of 82 °C, and pre-treatment time of 102 min, enhanced biogas and methane yields by 33.88% and 22% respectively in comparison with untreated SPW. The retention time in the bio-digester was also reduced from 22 days for untreated to 16 days for the thermochemically pre-treated SPW. Thermochemical pre-treatment of SPW which is rich in starch resulted in improved biogas and methane yield as well as a reduction in retention time, however, other pre-treatment methods for this waste could also be tried and the quality of the digestate should also be analysed.

## Declarations

### Author contribution statement

Chebet Catherine: Conceived and designed the experiments; Performed the experiments; Analyzed and interpreted the data.

Maurice Twizerimana: Contributed reagents, materials, analysis tools or data; Wrote the paper.

### Funding statement

This research did not receive any specific grant from funding agencies in the public, commercial, or not-for-profit sectors.

### Data availability statement

No data was used for the research described in the article.

### Declaration of interest’s statement

The authors declare no conflict of interest.

### Additional information

No additional information is available for this paper.
